# Toll-like receptor 4 monoclonal antibody attenuates lipopolysaccharide-induced acute lung injury in mice

**DOI:** 10.3892/etm.2014.1805

**Published:** 2014-06-24

**Authors:** ZHIJIE HE, XIAOTONG CHEN, SHOUPING WANG, ZIJUN ZOU

**Affiliations:** 1 Department of Critical Care Medicine, Sun Yat-sen Memorial Hospital, University of Sun Yat-sen, Guangzhou, Guangdong 510120, P.R. China; 2 Department of Anesthesiology, Sun Yat-sen Memorial Hospital, University of Sun Yat-sen, Guangzhou, Guangdong 510120, P.R. China

**Keywords:** Toll-like receptor 4 monoclonal antibody, acute lung injury, pulmonary edema, lung protection, rodents

## Abstract

Toll-like receptor 4 (TLR4) has an important role in the recognition of lipopolysaccharide (LPS) and in the activation of the inflammatory cascade. In the present study, the effect of TLR4 monoclonal antibody (mAb) on LPS-induced acute lung injury (ALI) was investigated in mice. A total of 45 male BALB/c mice were randomly divided into three groups, namely, the control (group C), sepsis (group S) and pretreatment groups (group P). Mice in group P were intraperitoneally treated with TLR4 mAb 1 h prior to the intraperitoneal administration of LPS. Following treatment with LPS for increasing times periods in groups S and P, the mRNA expression level of TLR4 in the lung tissue and the expression of inflammatory factors in the serum were analyzed by quantitative polymerase chain reaction and enzyme-linked immunosorbent assays, respectively. The degree of pulmonary edema, expressed as (wet weight - dry weight)/wet weight, as well as the lung injury scores, observed using a light microscope, were also analyzed. The results demonstrated that intraperitoneal administration of LPS in mice increased the mRNA expression levels of TLR4, the secretion of inflammatory factors in the serum, the degree of pulmonary edema and the lung injury score in a time-dependent manner. However, pretreatment with TLR4 mAb effectively attenuated the increased mRNA expression of TLR4 and the overproduction of inflammatory factors to correct the pulmonary edema and the elevated lung injury score induced by LPS. Therefore, TLR4 plays a critical role in LPS-induced ALI, and the TLR4 mAb decreases the secretion of inflammatory factors and attenuates the degree of pulmonary edema, thereby protecting the lungs from LPS-induced ALI.

## Introduction

Sepsis is a complex clinical syndrome that is caused by a harmful host response to infection. Numerous advances have been made in antibiotic therapy and supportive care; however, sepsis remains a major cause of mortality in intensive care units. The lung is the most vulnerable organ during sepsis (1). During the development of sepsis, bacterial components, such as lipopolysaccharide (LPS), may activate an inflammatory cascade, which results in the release of inflammatory mediators. The overproduction of inflammatory mediators induces endothelial and epithelial injury, vascular leakage, edema and vasodilatation, subsequently causing the development of acute lung injury (ALI) and acute respiratory distress syndrome (ARDS) (2,3). Previous studies have shown that inflammatory mediators have a key function in the pathogenesis of ALI/ARDS. ALI is characterized by a local inflammatory response, and Toll-like receptor 4 (TLR4) has an important function in the activation of innate immunity via recognizing LPS (4,5). Based on the key role of inflammatory mediators in the pathogenesis of ALI, recent treatments have been aimed at modulating TLR4 signaling and alleviating nonspecific inflammatory reactions that may result in potential therapeutic advantages for ALI.

As an essential receptor for LPS signaling, TLR4 may trigger the activation of an extracellular signaling pathway and result in the upregulation of inflammatory mediators (1,6). A previous study demonstrated that therapeutic antagonism of TLR4 signaling protects against ALI (7). To neutralize LPS signaling, the specific TLR4 monoclonal antibody (mAb) was used in the present study to evaluate the effect on an experimental model of ALI. It was hypothesized that suppressing TLR4-associated production of inflammatory mediators by pretreatment with TLR4 mAb may be a promising therapeutic strategy for the treatment of ALI.

## Materials and methods

### Animals

A total of 45 male BALB/c mice, weighing between 18 and 20 g, were obtained from the Guangdong Province Medical Experiments Animal Center. Mice were provided with free access to water and standard rodent chow, and were housed in pathogen-free cages. The study was performed in accordance with the recommendations in the Guide for the Care and Use of Laboratory Animals of the National Institutes of Health. The animal use protocol was reviewed and approved by the Institutional Animal Care and Use Committee of Sun Yat-sen Memorial Hospital of the University of Sun Yat-sen (Guangzhou, China).

### Experimental protocol

Mice were divided into three groups: Control (group C), sepsis (group S) and pretreatment (group P) groups. Mice in the P and S groups were intraperitoneally injected with 10 mg/kg LPS (Sigma-Aldrich, St. Louis, MO, USA) to establish an ALI model (8). Group P mice were also intraperitoneally injected with TLR4 mAb (5 μg/g; GeneTex, Irvine, CA, USA) 1 h prior to the injection of LPS. The mRNA expression levels of TLR4 in the lung tissue, as well as the serum expression levels of tumor necrosis factor (TNF)-α and interleukin (IL)-6, the water content of the lung and the pathomorphological changes in the lung, were detected after 6, 12 and 24 h.

### Analysis of inflammatory mediators in the serum

Mice were sacrificed via an intraperitoneal injection of 120 mg/kg pentobarbital, and blood samples were collected from the right atrium. The expression levels of TNF-α and IL-6 in the serum were then determined using an enzyme-linked immunosorbent assay (ELISA; Biolegend, San Diego, CA, USA), in accordance with the manufacturer’s instructions.

### Lung histology

Following euthanasia, the lungs were excised from the mice by opening the chest via median sternotomy. The right inferior lobe was removed and fixed in 10% buffered formalin for 24 h. Hematoxylin and eosin-stained sections were prepared using standard techniques as described by Szaka *et al* (9). The degree of microscopic injury was scored based on the following variables: Hemorrhage, edema, exudation, necrosis, congestion, neutrophil infiltration and atelectasis. The severity of injury was judged based on the following criteria (10): No injury, 0; injury to 25% of the field, 1; injury to 50% of the field, 2; injury to 75% of the field, 3; and diffuse injury, 4. The ultimate score was obtained by adding the aforementioned scores. A pathologist, who was blinded to the experimental protocol, provided a score for each variable based on the severity of injury (11,12).

### Water content of lung

Following euthanasia, the right middle lobe was excised from each mouse. The wet weight of the lung was measured using an electronic scale and then desiccated in an oven at 85°C for 48 h to determine the dry weight. The water content was obtained using the following equation: Water content (%) = (wet weight - dry weight)/wet weight × 100%.

### Expression of TLR4 mRNA in the lung tissue

The mRNA expression levels of TLR4 were analyzed by quantitative polymerase chain reaction. Total RNA was isolated from the lung tissue using TRIzol reagent (Invitrogen Life Technologies, Carlsbad, CA, USA) and subsequently reverse-transcribed into cDNA using Superscript II RNase H-Reverse Transcriptase (Invitrogen Life Technologies). The sequence-specific primers used were as follows: β-actin (sense), 5′-TACAGCTTCACCACCACAGC-3′ and antisense, 5′-AAGGAAGGCTGGAAGAGAGC-3′; TLR4 sense, 5′-TGCAATGTGAGCATTGATGA-3′ and antisense, 5′-TGACACCATTGAAGCTGAGG-3′.

### Statistical analysis

All data are presented as the mean ± standard deviation. GraphPad Prism 4.0 software (GraphPad Software, Inc., San Diego, CA, USA) was used for statistical analysis. Data were analyzed using one-way analysis of variance (ANOVA) by comparing the inter-group results and via two-way ANOVA by comparing the intra-group results. P<0.05 was considered to indicate a statistically significant difference.

## Results

### Water content in the lung tissue

As shown in Table I, the changes in the water content in the lungs of the mice in groups C, S and P were not statistically different at 6 h (P>0.05). However, when the LPS treatment time was increased, the water content in the lung tissue increased by ~13 and 16% at 12 and 24 h, respectively, as compared with group C. This result is in accordance with the changes observed in ALI. Pretreatment with TLR4 mAb prior to treatment with LPS was found to correct the LPS-induced increase in the water content of the lung tissue (P<0.05).

### Expression of TNF-α in the serum

To evaluate the effect of TLR4 mAb on an LPS-induced inflammatory mediator, the expression of TNF-α in the serum was measured using ELISA. As shown in Table II, the expression of TNF-α in group S significantly increased by 230 pg/ml maximally at 6 h (P<0.05) when compared with group C. Compared with group S, pretreatment with TLR4 mAb prior to LPS treatment reduced the expression of TNF-α induced by LPS maximally by 100 pg/ml at 6 h (P<0.05).

### Expression of IL-6 in the serum

Changes in the serum expression levels of IL-6 were also investigated, and the results were similar to those observed with TNF-α. As shown in Table III, the expression levels of IL-6 in group S significantly increased by 310 pg/ml maximally at 24 h (P<0.05) when compared with group C, and pretreatment with TLR4 mAb also effectively inhibited the increase in TNF-α expression induced by LPS (P<0.05). These results indicated that pretreatment with TLR4 mAb may correct the expression of LPS-induced inflammatory mediators.

### Expression of TLR4 mRNA in the lung tissue

**Figure 1:**
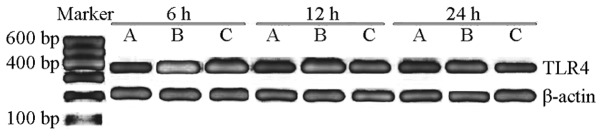
Product electropherogram of TLR4 mRNA, as determined by quantitative polymerase chain reaction. A, group C; B, group S; C, group P; TLR, Toll-like receptor; C, control; S, sepsis; P, pretreatment.

The mRNA expression level of TLR4 in group C was 0.304±0.03. The mRNA expression level of TLR4 in group S significantly increased compared with that in group C (P<0.05), and the peak value was observed at 6 h (1.697±0.090), which then gradually decreased after 12 and 24 h (1.280±0.083 and 0.913±0.093, respectively). However, the expression levels of TLR4 mRNA at 6, 12 and 24 h in group P were 1.201±0.053, 0.921±0.049 and 0.404±0.051, respectively, thus, were significantly decreased compared with group S (P<0.05; Table IV, Fig. 1). These observations indicated that TLR4 mAb may decrease the mRNA expression of TLR4 in the lung tissue of sepsis mice.

### Pathological examination of the lung tissue using light microscopy

**Figure 2:**
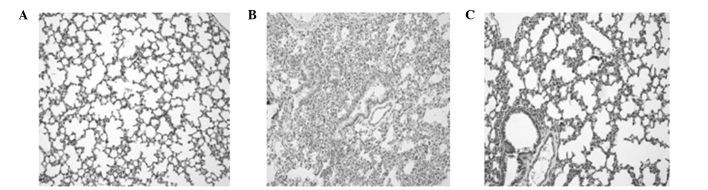
Changes in the histology and lung injury scores after 24 h in groups (A) C (lung injury score, 1), (B) S (lung injury score, 10) and (C) P (lung injury score, 4). Hematoxylin and eosin staining; magnification, ×200. C, control; S, sepsis; P, pretreatment.

The pulmonary organizational structure of group C was normal. However, in group S at 6 and 12 h, the dilation of the alveolus was not uniform since a number of the alveoli had collapsed. Incrassation of the alveoli septum, neutrophil infiltration and enlargement and congestion of the capillaries were also observed. The extent of the pathological changes was aggravated with increasing duration. In group S at 24 h, in addition to the aforementioned pathological changes, airway trauma, lung edema and local lung hemorrhage were observed. The pathological changes in group P were significantly improved compared with those in group S (Fig. 2).

### Ultrastructural changes of the lung tissue observed using electron microscopy

**Figure 3:**
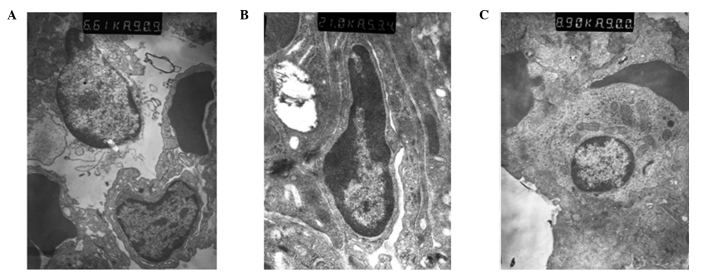
Ultrastructural changes of the lung tissue observed with electron microscopy after 12 h in groups (A) C, (B) S and (C) P. C, control; S, sepsis; P, pretreatment.

In group C, the structure of the lung tissue and alveolar were normal and clear, and edema and inflammatory changes were observed. However, ultrastructural injury in the lung tissue was observed in group S. In addition, alveolar epithelial cell vacuolization and organelle swelling, dissolution and necrosis were observed. The nuclei of the cells were twisted, and sections of the chromatin were dissolved and had migrated to the edge of the cells. Alveolar wall thickening and capillary expansion, as well as pulmonary microvascular basement membrane thickening, were also observed. Over time, rupture with widened alveolar septa and pulmonary interstitial edema were observed, along with the infiltration of the alveolar space by numerous red blood cells and neutrophil granulocytes. The ultrastructural injury in group P was milder compared with group S, in which pulmonary interstitial edema was not observed and cell infiltration was reduced (Fig. 3).

## Discussion

TLRs were identified in the late 1990′s as primary sensors of microbial infection, and their identification led to significant advances (13), including that TLRs were broadly involved in transducing sensory information in bacteria and TLR4 activated NF-κB triggers the production of several inflammatory cytokines. Currently, 12 members of the TLR family have been identified in mammalian cells (14). Among these, TLR4 detects the presence of gram-negative bacteria through the recognition of the lipid A moiety of LPS (15,16). TLR4 is a type I integral membrane glycoprotein with a cytoplasmic signaling domain that is homologous to the signaling domain of the IL-1 receptor (IL-1R), known as the Toll/IL-1R homology (TIR) domain (17). Following ligand binding, TLR4 recruits the TIR-domain-containing adaptor molecules to the TIR domain. Myeloid differentiation factor 88 (MyD88) is an important adaptor molecule. Upon stimulation, MyD88 associates with the cytoplasmic domain of TLR4 and subsequently recruits IL-1R-associated kinase 1 (IRAK-1) and IRAK-4 (18–20), which mediate the activation of the transcriptional factor, nuclear factor (NF)-κB, leading to the induction of inflammatory mediators. The initiation of the innate immune response through TLR4 triggers an inflammatory cascade that is the primary cause of harmful conditions, including ALI. Therefore, to suppress the inflammatory cascade effectively, we hypothesized that the inhibition of a point upstream of the TLR4 signaling pathway may be a better approach than inhibiting each mediator. Abraham (19) used a TNF-α mAb to treat septic shock, while Fisher (20) used an IL-1R antagonist in the treatment of patients with sepsis syndrome; however, the results were not satisfactory.

Previous studies have shown that the therapeutic antagonism of TLR4 signaling provides protection against ALI. Recently, Shirey *et al* (7) found that Eritoran (E5564), an extremely potent TLR4 antagonist, exhibits a highly protective effect when administered therapeutically to mice with influenza-induced ALI (7). In addition, it has previously been found that TLR4 mAb may function as a TLR4 antagonist and reduce the expression of IL-1β in LPS-stimulated decidual cells (21,22). Furthermore, a previous study demonstrated that TLR4 mAb reduces LPS-induced cytokine production by inhibiting the NF-κB pathway in cells (23). ALI is characterized by a local inflammatory response, and the potential efficacy of TLR4 mAb to treat ALI is based on the hypothesis that TLR4 mAb suppresses the TLR4-associated inflammatory cascade of ALI. In the present study, TLR4 mAb was shown to exhibit a protective effect on LPS-induced ALI in mice. The expression levels of TNF-α and IL-6 decreased, whilst lung histology and edema were markedly improved. However, the underlying mechanisms require further investigation.

In the present study, significantly increased serum levels of TNF-α and IL-6 were observed in group S when compared with those in group C, indicating that TNF-α and IL-6 have an important role in LPS-induced ALI. TNF-α is derived from activated macrophages and functions via specific cell membrane-bound receptors. Injecting TNF-α into experimental animals causes a syndrome similar to septic shock (1,24,25). TNF-α is released during the first 30–90 min following exposure to LPS, which in turn activates a secondary level of the inflammatory cascade, including cytokines, lipid mediators and reactive oxygen species (1). IL-6 is produced by a wide range of cells, including macrophages and endothelial cells, in response to stimulation by factors, such as endotoxins and TNF-α (26–28). IL-6 is an important factor that amplifies the inflammatory reaction and stimulates the synthesis of acute phase protein (29,30) Circulating levels of IL-6 are excellent predictors of the severity of ALI/ARDS of various etiologies (31). In the present study, significantly decreased levels of TNF-α and IL-6 were observed in group P when compared with those in group S. The inhibition of TLR4 signaling by TLR4 mAb resulted in the suppression of TNF-α and IL-6, which resulted in low-level lung edema and injury. TLR4 signaling is a critical activator of immune defense during infection, and the effective termination of TLR signaling is essential to prevent detrimental systemic effects, including septic shock (32). Therefore, the signaling mechanism of TLR4 mAb, the regulation of TLR4 expression on the cell surface and the transcriptional induction of negative regulators, including IL-1R-associated kinase and suppressor of cytokine signaling 1, requires further investigation (33).

In conclusion, TLR4 plays a critical role in LPS induced-ALI. TLR4 mAb reduces the secretion of inflammatory factors and attenuates the degree of pulmonary edema, thus, exhibits protective effects against LPS-induced ALI.

## Figures and Tables

**Table I:** Water content in the lung tissue of each group.

Parameter	6 h	12 h	24 h
Group C (%)	72.47±2.89	71.32±4.13	71.20±3.11
Group S (%)	77.63±4.13	84.29±4.69^a^,^c^	87.23±5.12^a^,^c^,^d^
Group P (%)	76.65±5.32	78.35±3.61^a^,^b^	83.32±4.87^a^,^b^,^c^,^d^
F-value	2.09	13.32	17.55

Results are expressed as the mean ± standard deviation (n=5).

a P<0.05, vs. group C;

b P<0.05, vs. group S;

c P<0.05, vs. intra-group 6 h;

d P<0.05, vs. intra-group 12 h.

C, control; S, sepsis; P, pretreatment. n is the number of mice used in each group.

**Table II:** Serum expression levels of TNF-α in each group.

Parameter	6 h	12 h	24 h
Group C (pg/ml)	29.45±5.25	33.13±3.88	34.08±4.12
Group S (pg/ml)	259.12±19.81^a^	186.21±13.75^a^,^c^	115.89±17.91^a^,^c^,^d^
Group P (pg/ml)	156.85±14.11^a^,^b^	112.43±14.33^a^,^b^,^c^	81.45±10.58^a^,^b^,^c^,^d^
F-value	324.96	214.53	56.21

Results are expressed as the mean ± standard deviation (n=5).

a P<0.05,vs. group C;

b P<0.05, vs. group S;

c P<0.05, vs. intra-group 6h;

d P<0.05, vs. intra-group 12h.

TNF, tumor necrosis factor; C, control; S, sepsis; P, pretreatment. n is the number of mice used in each group.

**Table III:** Serum expression levels of IL-6 in each group.

Parameter	6 h	12 h	24 h
Group C (pg/ml)	84.83±5.74	86.52±7.37	84.96±10.06
Group S (pg/ml)	235.75±32.26^a^	300.64±13.65^a^,^c^	394.58±13.55^a^,^c^,^d^
Group P (pg/ml)	157.93±18.41^a^,^b^	204.58±15.40^a^,^b^,^c^	308.59±14.11^a^,^b^,^c^,^d^
F-value	60.45	360.78	791.18

Results are expressed as the mean ± standard deviation (n=5).

a P<0.05, vs. group C;

b P<0.05, vs. group S;

c P<0.05, vs. intra-group 6 h;

d P<0.05, vs. intra-group 12 h.

IL, interleukin; C, control; S, sepsis; P, pretreatment. n is the number of mice used in each group.

**Table IV:** mRNA expression levels of TLR4/β-actin in each group.

Parameter	6 h	12 h	24 h
Group C (%)	0.304±0.031	0.315±0.022	0.309±0.025
Group S (%)	1.697±0.090^a^	1.280±0.083^a^,^c^	0.913±0.093^a^,^c^,^d^
Group P (%)	1.201±0.053^a^,^b^	0.921±0.049^a^,^b^,^c^	0.404±0.051^a^,^b^,^c^,^d^
F-value	70.21	58.35	36.48

Results are expressed as the mean ± standard deviation (n=5).

a P<0.05, vs. group C;

b P<0.05, vs. group S;

c P<0.05, vs. intra-group 6 h;

d P<0.05, vs. intra-group 12 h.

TLR, Toll-like receptor; C, control; S, sepsis; P, pretreatment.
